# Prognostic value of volumetric metabolic parameters measured by [^18^F]Fluorodeoxyglucose-positron emission tomography/computed tomography in patients with small cell lung cancer

**DOI:** 10.1186/1470-7330-14-2

**Published:** 2014-04-22

**Authors:** Soo Bin Park, Joon Young Choi, Seung Hwan Moon, Jang Yoo, Hojoong Kim, Yong Chan Ahn, Myung-Ju Ahn, Keunchil Park, Byung-Tae Kim

**Affiliations:** 1 Department of Nuclear Medicine, Samsung Medical Center, Sungkyunkwan University School of Medicine, 50 Irwon-dong, Gangnam-gu, Seoul 135-710, Korea; 2 Division of Pulmonary and Critical Care, Department of Medicine, Samsung Medical Center, Sungkyunkwan University School of Medicine, Seoul, Korea; 3 Department of Radiation Oncology, Samsung Medical Center, Sungkyunkwan University School of Medicine, Seoul, Korea; 4 Division of Hemato-Oncology, Department of Medicine, Samsung Medical Center, Sungkyunkwan University School of Medicine, Seoul, Korea

**Keywords:** Small cell lung cancer, [^18^F]FDG-PET/CT, Metabolic tumor volume, Total lesion glycolysis, Prognosis

## Abstract

**Background:**

We evaluated the prognostic value of volume-based metabolic positron emission tomography (PET) parameters in patients with small cell lung cancer (SCLC) compared with other factors.

**Methods:**

The subjects were 202 patients with pathologically proven SCLC who underwent pretreatment ^18^F-fluorodeoxyglucose (FDG) PET/computed tomography (CT). Volumetric metabolic parameters of intrathoracic malignant hypermetabolic lesions, including maximum and average standardized uptake value, sum of metabolic tumor volume (MTV), and sum of total lesion glycolysis (TLG) were measured.

**Results:**

164 patients had died during follow-up (median 17.4 months) and median overall survival was 14 months. On univariate survival analysis, age, stage, treatment modality, sum of MTV (cutoff = 100 cm^3^), and sum of TLG (cutoff = 555) were significant predictors of survival. There was a very high correlation between the sum of MTV and the sum of TLG (r = 0.963, *P* < 0.001). On multivariate survival analysis, age (HR = 1.04, *P* < 0.001), stage (HR = 2.442, *P* < 0.001), and sum of MTV (HR = 1.662, *P* = 0.002) were independent prognostic factors. On subgroup analysis based on limited disease (LD) and extensive disease (ED), sum of MTV and sum of TLG were significant prognostic factors only in LD.

**Conclusion:**

Both sum of MTV and sum of TLG of intrathoracic malignant hypermetabolic lesions are important independent prognostic factors for survival in patients with SCLC, in addition to age and clinical stage. However, it may be more useful in limited disease rather than in extensive disease.

## Background

In 2008, an estimated 1,608,800 new lung cancer cases were diagnosed globally, and there were 1,378,400 estimated deaths [1]. Small cell lung cancer (SCLC), which accounts for 13% of all lung cancer [2], is distinct from non-SCLC due to its rapid doubling time, high growth fraction, and early development of widespread metastases. However, despite high initial responses to therapy, most patients die from recurrent disease [3]. Clinical stage at initial presentation is the most powerful prognostic factor of SCLC, with other factors including performance status, age, gender, lactate dehydrogenase (LDH), and albumin [4].

The simple two-stage system of the Veterans Administration Lung Study Group is generally accepted in SCLC, which classifies cases as limited disease (LD; primary tumor and nodal involvement limited to one hemithorax) and extensive disease (ED; inoperable patients who cannot be classified as having LD) depending mainly on whether all known tumors could be treated within a single radiotherapy portal [5]. The standard of care in patients of good performance status with LD is concurrent chemotherapy and radiotherapy followed by prophylactic cranial irradiation in those responding to treatment. The mainstay of treatment for ED is palliative chemotherapy. Because of the differences in treatment strategies and prognoses of patients with LD and ED, accurate staging to select the appropriate treatment for an individual patient is of paramount importance [6]. However, current prognostic evaluations are still controversial, and the two-stage system is unsatisfactory in terms of predicting prognosis [5,7]. For example, ipsilateral pleural effusion and contralateral mediastinal or supraclavicular lymph node metastases are neither precisely defined nor uniformly handled by investigators, which may have high prognostic impact [5]. Therefore, more discriminative prognostic markers are necessary to select the appropriate treatment and to properly predict treatment outcomes and survival.

In SCLC, [^18^F]fluorodeoxyglucose-positron emission tomography (FDG-PET) has emerged as an essential imaging tool for staging, impact on patient management, and early evaluation of treatment response [8-15], and FDG-PET findings have been suggested as useful prognostic indicators [16-19]. While volumetric metabolic parameters, such as metabolic tumor volume (MTV) or total lesion glycolysis (TLG) have been reported to be independent prognostic factors in other types of malignancies [19-27], only two studies have evaluated the volumetric metabolic parameters in patients with SCLC [19,26]. These studies used the volumetric parameters of all hypermetabolic lesions in the whole body, which may be time-consuming and not practical for routine practice. In addition, they used a fixed threshold standardized uptake value (SUV) of 2.5 or 3.0. In this setting, it may be difficult to measure the TLG or MTV of lesions within the organs with relatively high physiological FDG uptake, such as the brain, liver, or bone marrow. Another potential limitation of previous studies has been the relatively small number of subjects. Therefore, we evaluated the prognostic value of volumetric metabolic parameters measured in intrathoracic lesions by FDG-PET/computed tomography (CT) in patients with SCLC and compared them with other prognostic parameters.

## Methods

### Subjects

We retrospectively reviewed the medical records of all 219 patients with pathologically proven SCLC who underwent pretreatment with FDG-PET/CT at the Samsung Medical Center, Sungkyunkwan University School of Medicine, between May 2003 and December 2009. Among them, patients who refused the treatment and were not followed up after diagnosis in our institution were excluded from further analysis, and the remaining 202 patients, who underwent any kinds of therapy in our institution, were included. All patients with SCLC underwent complete blood cell counts and chemistry panels, CT of the chest and upper abdomen, brain magnetic resonance imaging, bronchoscopy and FDG-PET/CT as a staging workup. Bone scintigraphy, abdominal or neck CT scan, abdominal ultrasonography, and neck ultrasonography were performed when clinically indicated. All staging workups were completed before initial therapy. Our institutional review board approved the study protocol (Samsung Medical Center IRB).

### [^18^F] Fluorodeoxyglucose-positron emission tomography/computed tomography

All subjects fasted for at least 6 hours before the PET/CT scans. Blood glucose levels at the time of injection of FDG were lower than 200 mg/dl in all patients. PET/CT scans were performed on two different dedicated PET/CT scanners (Discovery LS or Discovery STE; GE Healthcare, Milwaukee, WI, USA). Among the 202 patients, scans in 140 were performed using the Discovery LS PET/CT scanner and scans in 62 patients were performed using the Discovery STE PET/CT scanner. No intravenous or oral contrast materials were used.

In the Discovery LS scanner, whole-body CT was performed using a continuous spiral technique with an 8-slice helical CT (140 kV, 40–120 mAs adjusted to the patient’s body weight, section width of 5 mm) 45 minutes after the injection of approximately 370 MBq FDG. After the CT scans were complete, emission scans were obtained from the thigh to the head for 4 minutes per frame in two-dimensional mode. Attenuation-corrected PET images (voxel size = 4.3 × 4.3 × 3.9 mm) were reconstructed using the CT data by an ordered-subsets expectation maximization algorithm (28 subsets, two iterations).

In the Discovery STE scanner, whole-body CT was performed using a continuous spiral technique with a 16-slice helical CT (140 kV, 30–170 mAs with an AutomA mode, section width of 3.75 mm) 60 minutes after the injection of FDG (5.5 MBq/kg). After the CT scans were complete, emission scans were obtained from the thigh to the head for 2.5 minutes per frame in three-dimensional (3D) mode. Attenuation-corrected PET images (voxel size = 3.9 × 3.9 × 3.3 mm) were reconstructed using CT data by a 3D ordered-subsets expectation maximization algorithm (20 subsets, two iterations). Commercial software (Advantage Workstation; GE Healthcare) was used to accurately coregister the separate CT and PET scan data.

### Measurement of positron emission tomography/computed tomography parameters

**Figure 1:**
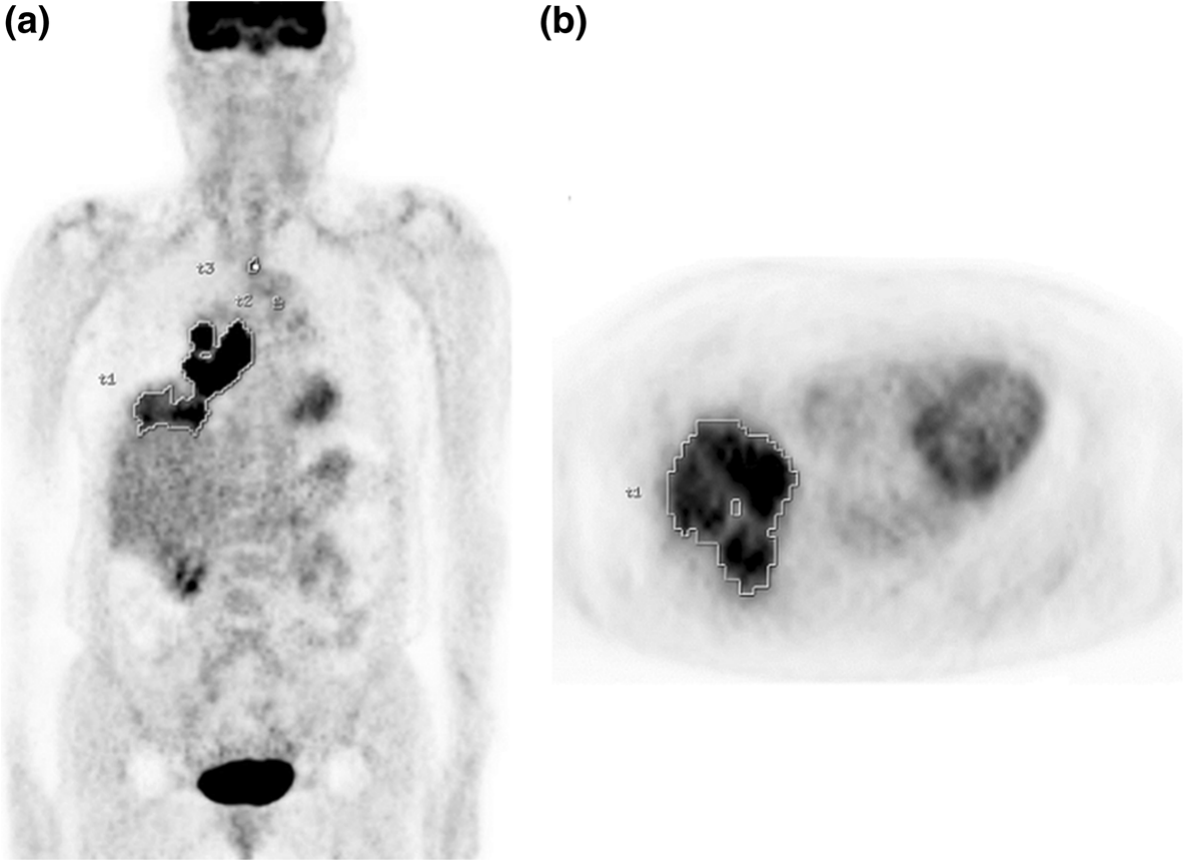
Representative coronal (a) and axial (b) positron emission tomography (PET) images of a patient with small-cell lung cancer showing automatically generated volumes of interest (VOIs) including all malignant hypermetabolic intrathoracic lesions.

Semiquantitative and volumetric analyses were performed using the PET VCAR software (GE Healthcare) on a GE Advantage Workstation 4.4 (GE Healthcare), which provides a convenient and automatic way to delineate the volume of interest (VOI) using an isocontour threshold method based on the SUV. We used a dynamic threshold SUV for determining the tumor boundary, which was derived from a 2-cm rectangular VOI placed at the right hepatic lobe (hepatic hilum level) [28]. The average SUV (SUV_avg_) of the liver VOI plus the two standard deviations (2 SDs) of each patient was adopted as the threshold SUV. We measured the PET parameters of all intrathoracic (lung, pleura, and mediastinum) malignant hypermetabolic lesions (Figure 1). Benign FDG-avid lesions, such as inflammatory lung lesions or reactive lymph nodes, were excluded from quantitative analysis based on histopathologic results or other imaging modalities. Using the above-mentioned threshold SUV, the software automatically generated the VOI of each hypermetabolic lesion and calculated the various metabolic PET parameters, including the maximum SUV (SUV_max_), SUV_avg_, and MTV of each hypermetabolic lesion. Total TLG was calculated by multiplying the SUV_avg_ by the MTV of each hypermetabolic lesion. The highest SUV_max_ among the intrathoracic malignant hypermetabolic lesions, and the sum of the MTV or TLG of all intrathoracic malignant hypermetabolic lesions, were adopted as prognostic variables.

### Treatment and clinical follow-up

None of the patients was surgically indicated. Patients with LD underwent concurrent chemoradiotherapy, and patients with ED underwent chemotherapy only as an initial therapy. The chemotherapy regimen, consisting of a platinum-based drug (cisplatin or carboplatin) with either etoposide or irinotecan, was administered every 3 weeks for 4–6 cycles. Chest irradiation was initiated on day 1 of the first or second cycle of chemotherapy with 2.1 Gy once daily in 25 fractions. Patients showing a complete response or partial response after chemoradiotherapy received prophylactic cranial irradiation, which consisted of 25 Gy in 10 fractions. The standard response evaluation consisted of a chest X-ray prior to each cycle and a chest CT every two cycles of chemotherapy. After completion of treatment, patients were clinically followed up with a chest CT every 3 months for 1 year, every 6 months in the following year, and every 6–12 months thereafter.

### Statistical analysis

Statistical analyses were performed using a commercial software program, PASW Statistics 18 (IBM Corporation, Armonk, NY, USA). The main end point of survival prediction was overall survival. Overall survival time was measured from the date of diagnosis to event or last clinical follow-up. The event for overall survival was defined as cancer- or treatment-related death. Survival curves were estimated using the Kaplan–Meier estimator. Maximally selected log rank statistics were used to determine the optimal cutoffs showing the best discrimination of survival curves for continuous variables. Prognostic significance of the volumetric metabolic PET parameters and other clinical variables were assessed by univariate and multivariate analyses using the Cox proportional hazards regression model, and an estimated hazard ratio (HR) with a 95% confidence interval (CI) was constructed. A *P* value < 0.05 was considered statistically significant.

## Results

The demographic and clinical characteristics of the 202 patients are presented in Table 1. The median age was 64.0 ± 8.8 years (range, 39–87 years). Follow-up data were available through to November 2011. At the time of analysis, 38 (19%) patients were still alive and 164 (81%) had died. Median clinical follow-up time was 17.4 months, with a range of 0–84 months. The median overall survival was 14 months (95% CI, 12.4–15.6 months).

**Table 1:** Demographic and clinical characteristics of the patients

**Characteristics**	**Number of patients (%)**
Age (years)	
< 65	100 (50)
≥ 65	102 (50)
Gender	
Men	179 (89)
Women	23 (11)
Clinical stage	
Limited	95 (47)
Extensive	107 (53)
Treatment modality	
Chemotherapy alone	117 (58)
Chemotherapy and radiotherapy	85 (42)
Performance status (ECOG)	
0.1	181 (90)
≥ 2	21 (10)
Albumin^a^	
< 3.5 g/dl	31 (15)
≥ 3.5 g/dl	170 (85)
Lactate dehydrogenase^b^	
< 480 IU/l	111 (64)
≥ 480 IU/l	63 (36)

ECOG, Eastern Cooperative Oncology Group.

^a^One missing data point.

^b^28 missing data points.

PET parameters for survival analysis included SUV_max_, SUV_avg_, sum of MTV, and the sum of TLG. For other clinical parameters, gender, age, clinical stage (LD vs. ED), treatment modality, performance status, serum albumin, and LDH were also assessed. Treatment modalities were categorized into chemotherapy alone and chemotherapy plus radiotherapy. The mean ± the standard deviation of SUV_max_, SUV_avg_, sum of MTV, and sum of TLG were 11.8 ± 4.3, 5.1 ± 1.0, 150.4 ± 169.9 cm^3^, and 788.3 ± 826.5, respectively. According to the optimal cutoff value for serum albumin (3.5 g/dl), LDH (480 IU/l), SUV_max_ (12.0), SUV_avg_ (5.0), sum of MTV (100 cm^3^), and sum of TLG (555), patients were divided into two groups. The smaller MTV group consisted of 92 patients and the larger MTV group of 110 patients; the smaller TLG group consisted of 95 patients and the larger TLG group of 107 patients.

**Figure 2:**
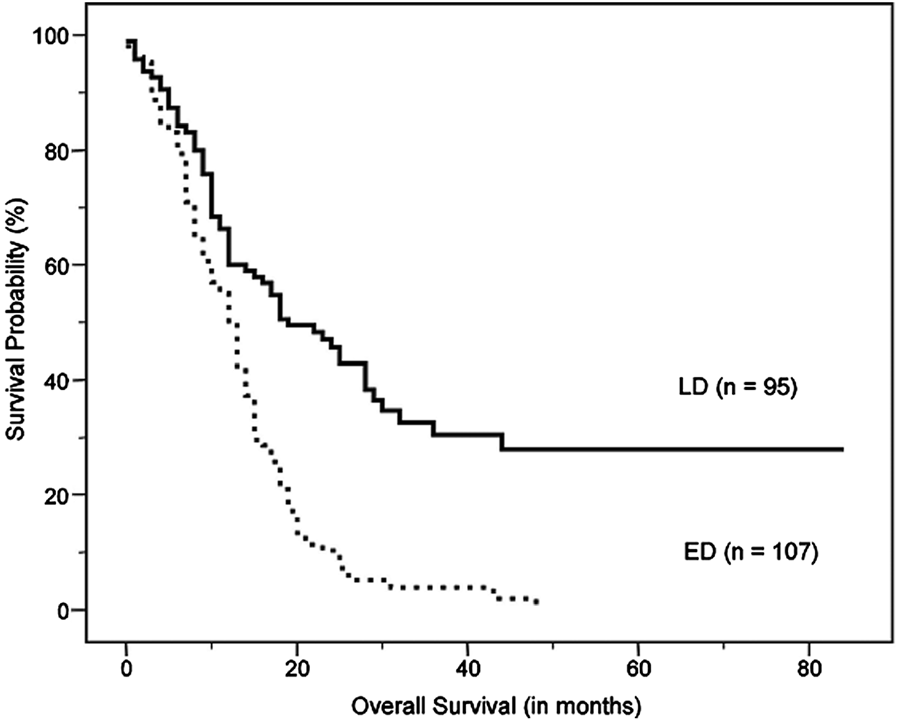
Kaplan–Meier curve for overall survival according to clinical stage, which shows significantly worse outcomes in extensive disease (ED) than in limited disease (LD) (median survival, 12.0 ± 0.7 months vs. 19.0 ± 3.3 months, P < 0.001).

**Figure 3:**
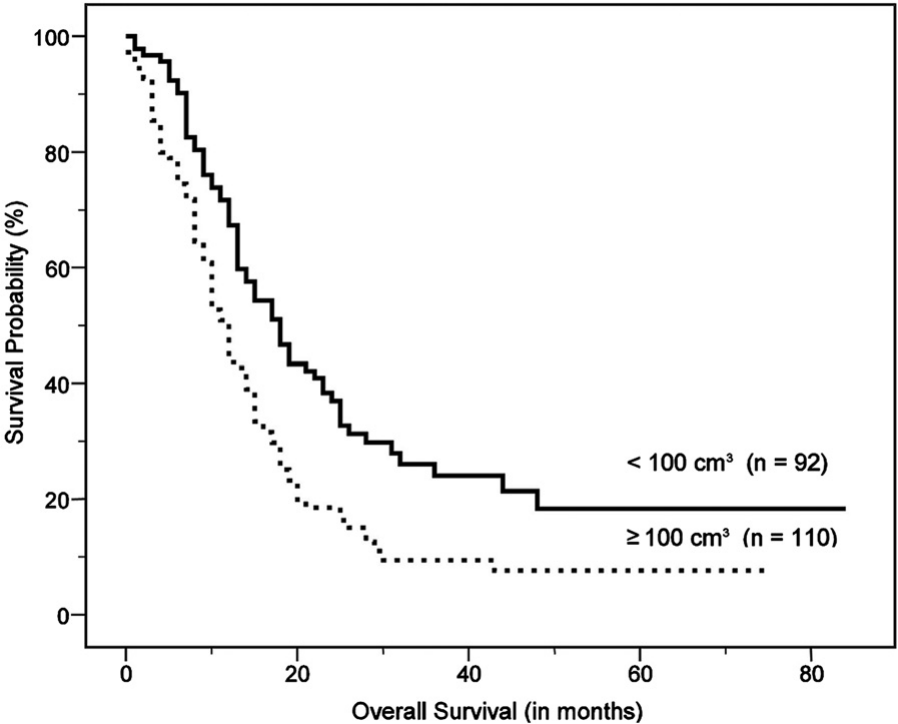
Kaplan–Meier curve for overall survival according to the sum of metabolic tumor volume (MTV) of malignant hypermetabolic intrathoracic lesions, which shows significantly worse outcomes in high MTV than in low MTV (median survival, 12.0 ± 0.8 months vs. 18.0 ± 1.8 months, P < 0.001).

**Figure 4:**
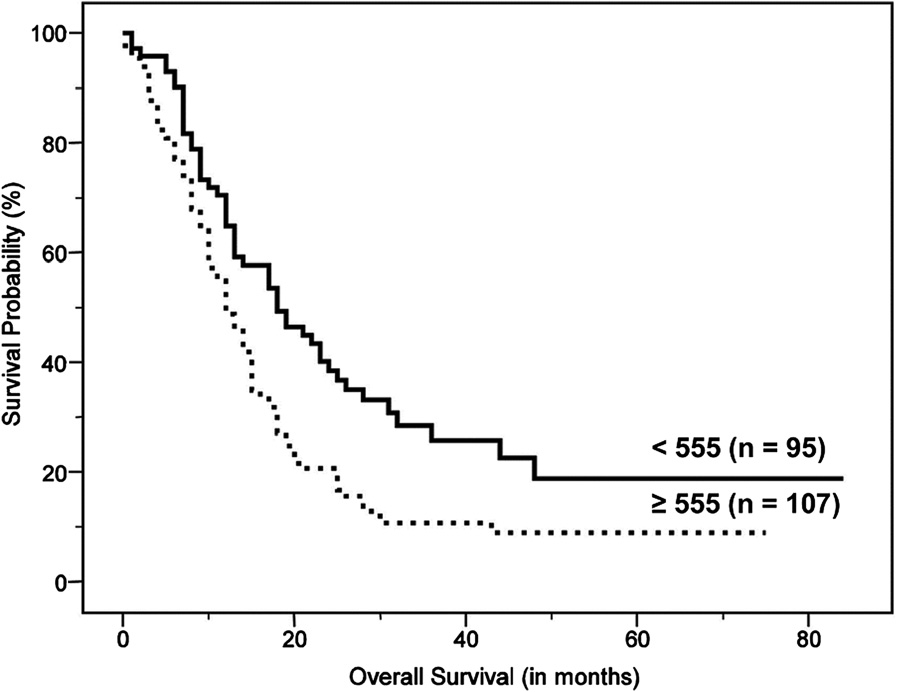
Kaplan–Meier curve for overall survival according to the sum of total lesion glycolysis (TLG) of malignant hypermetabolic intrathoracic lesions, which shows significantly worse outcomes in high TLG than in low TLG (median survival, 12.0 ± 1.1 months vs. 18.0 ± 2.0 months, P = 0.001).

On univariate analysis, age, clinical stage, treatment modality, sum of MTV, and sum of TLG were significant predictors of survival (Table 2). Figures 2, 3 and 4 show the Kaplan–Meier curves for overall survival according to clinical stage, sum of MTV, and sum of TLG, respectively. In other words, old age, high clinical stage, chemotherapy only, high sum of MTV, and high sum of TLG were associated with poor survival outcomes. There were no significant differences in survival according to gender, performance status, serum albumin, LDH, SUV_max_, or SUV_avg_. On multivariate analysis, age, clinical stage, and sum of MTV were independent prognostic factors (Table 3). Because there was a very high correlation between the sum of MTV and the sum of TLG (*r* = 0.963, *P* < 0.001), additional multivariate analysis was performed twice excluding the sum of MTV or sum of TLG as a variable. The results revealed that both sum of MTV (HR = 1.626, p = 0.003) or sum of TLG (HR = 1.548, p = 0.007) was a significant independent prognostic factor in each analysis.

**Table 2:** Results of the univariate survival analysis

**Variable**	**HR**	**95% CI**	***P***
Age (1-year increase)	1.031	1.012–1.050	0.001
Clinical stage			
Extensive (*n* = 107) vs. limited (*n* = 95)	2.446	1.761–3.397	< 0.001
Treatment modality			
CTX only (*n* = 117) vs. CTX and RTX (*n* = 85)	2.181	1.573–3.023	< 0.001
Sum of MTV			
10-cm^3^ increase	1.009	1.003–1.015	0.004
≥ 100 cm^3^ (*n* = 110) vs. < 100 cm^3^ (*n* = 92)	1.748	1.277–2.392	< 0.001
Sum of TLG			
10-unit increase	1.002	1.001–1.004	0.001
≥ 555 (*n* = 107) vs. < 555 (*n* = 95)	1.697	1.242–2.318	0.001

HR, hazard ratio; CI, confidence interval; CTX, chemotherapy; RTX, radiotherapy; MTV, metabolic tumor volume; TLG, total lesion glycolysis.

**Table 3:** Results of the multivariate survival analysis

**Variable**	**HR**	**95% CI**	***P***
Age (1-year increase)	1.04	1.02–1.06	< 0.001
Clinical stage			
Extensive vs. limited	2.442	1.742–3.425	< 0.001
Sum of MTV			
≥ 100 cm^3^ vs. < 100 cm^3^	1.662	1.204–2.295	0.002

HR, hazard ratio; CI, confidence interval; MTV, metabolic tumor volume.

**Figure 5:**
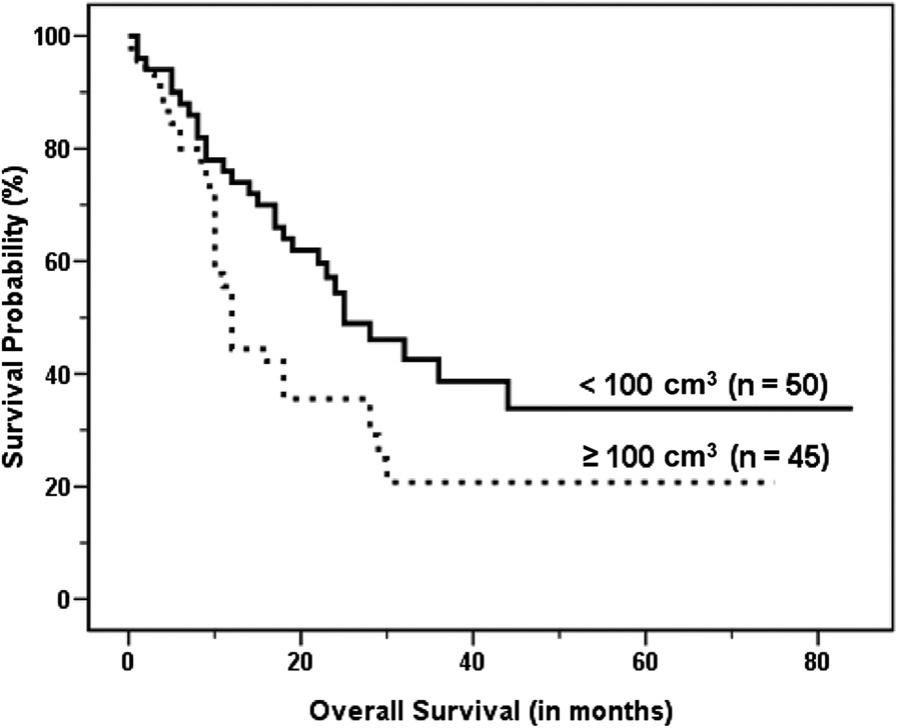
Kaplan–Meier curve for overall survival according to the sum of metabolic tumor volume (MTV) of malignant hypermetabolic intrathoracic lesions in limited disease (LD), which shows significantly worse outcomes in high MTV than in low MTV (median survival, 12.0 ± 1.1 months vs. 25.0 ± 4.9 months, P = 0.036).

**Figure 6:**
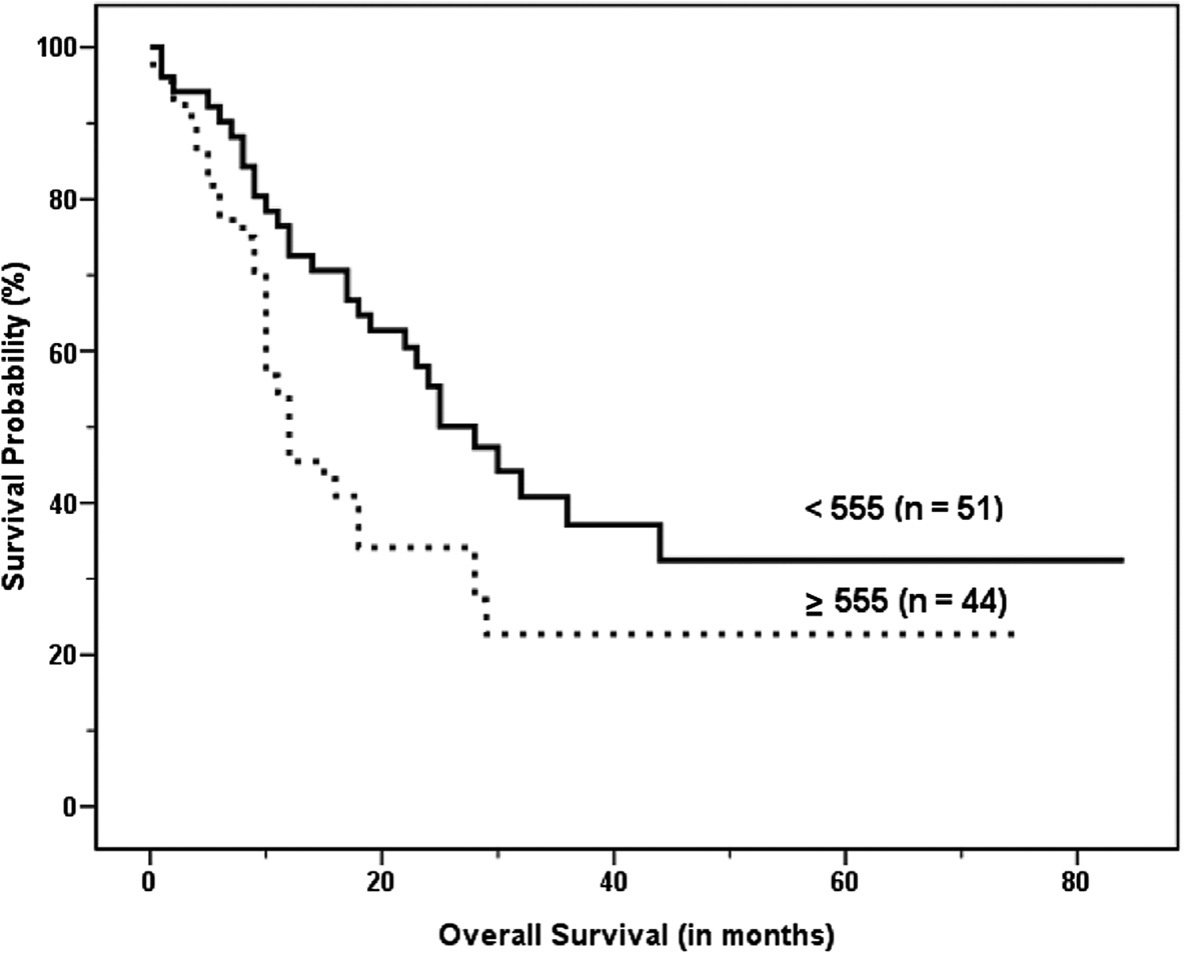
Kaplan–Meier curve for overall survival according to the sum of total lesion glycolysis (TLG) of malignant hypermetabolic intrathoracic lesions in limited disease (LD), which shows significantly worse outcomes in high TLG than in low TLG (median survival; 12.0 ± 1.3 months vs. 28.0 ± 4.0 months, P = 0.03).

Subgroup survival analysis was performed according to the stage. In LD, only the sum of MTV and sum of TLG were significant prognostic factors in the univariate analysis (Table 4; Figures 5 and 6). There were no significant differences in survival according to age, treatment modality, gender, performance status, serum albumin, LDH, SUV_max_, or SUV_avg_. In ED, age was the only significant prognostic factor in the univariate analysis (*P* < 0.001). There were no significant differences in survival according to treatment modality, gender, performance status, serum albumin, LDH, SUV_max_, SUV_avg_, sum of MTV (*P* = 0.071), or sum of TLG (*P* = 0.081).

**Table 4:** Results of the univariate survival analysis in limited disease

**Variable**	**HR**	**95% CI**	***P***
Sum of MTV			
10-cm^3^ increase	1.025	0.999–1.050	0.055
≥ 100 cm^3^ (*n* = 45) vs. < 100 cm^3^ (*n* = 50)	1.779	1.072–2.959	0.036
Sum of TLG			
10-unit increase	1.004	1.000–1.009	0.051
≥ 555 (*n* = 44) vs. < 555 (*n* = 51)	1.815	1.094–3.012	0.03

HR, hazard ratio; CI, confidence interval; MTV, metabolic tumor volume; TLG, total lesion glycolysis.

## Discussion

Several SCLC studies have evaluated the prognostic significance of FDG-PET. These studies suggested that high SUV_max_ of the tumor and remaining FDG uptake after treatment were poor prognostic factors [16-19]. Since SUV is a semiquantitative index for FDG uptake of a tumor, it gives one possibility for obtaining metabolic information. However, it remains unclear whether SUV is an independent prognostic factor when compared with stage [19,26]. In the current study, SUV_max_ was not a significant independent prognostic factor for survival on univariate analysis (*P* = 0.168). Our results are consistent with those of previous reports that have found that SUV_max_ is not an independent prognostic factor [19,26]. This can be partly explained by the partial volume effect and the dependence of SUV_max_ on tumor size. SUV is a single voxel measurement, with the highest radiotracer concentration within the VOI, and so it may not reflect the heterogeneous nature of the tumor and it is easily affected by statistical noise and voxel size [29]. Different from SUV_max_, which only represents single voxels, MTV and TLG represent the extent of FDG uptake of the whole tumor, and have been suggested as better prognostic indicators than SUV_max_ for clinical outcomes in other types of malignancies [20,25].

This is the largest study of volumetric PET parameters as prognostic factors for survival in patients with SCLC to date (202 patients). On univariate analysis, both the sum of MTV and sum of TLG were significant prognostic factors, along with age, clinical stage, and treatment modality. However, on multivariate analysis, only the sum of MTV was an independent significant prognostic factor along with age and clinical stage. When multivariate analysis was performed twice including only either the sum of MTV or sum of TLG because of high correlation between MTV and TLG, the analysis revealed that both were significant independent prognostic factors with nearly the same HRs and *P* values. As the measurement of MTV is simpler and MTV is a better independent prognostic factor for survival as revealed on multivariate analysis, the sum of MTV of the intrathoracic malignant hypermetabolic lesions may be more suitable in routine clinical practice for predicting survival prognosis in SCLC. Zhu et al. [19] reported higher HR in MTV than in the integrated SUV (iSUV, an analog to our TLG).

In subgroup analysis according to the stage, the volumetric PET parameters were significant prognostic factors for survival in patients with LD, but not in patients with ED, which may result from the higher event rate of patient with ED (98%) compared to LD (64%). Therefore, the volumetric PET parameters may be more useful as prognostic factors in patients with LD rather than ED. The median survival in LD with the high MTV (12.0 ± 1.1 months) and high TLG (12.0 ± 1.3 months) was comparable to that of ED (12.0 ± 0.7 months). Thus, the LD group with high metabolic parameters may be treated differently from the group with low metabolic parameters, and may deserve close follow-up for surveillance. In other words, the LD group with high metabolic parameters may be suggested to be treated as ED. A further prospective study is warranted.

Zhu et al. [19] demonstrated that MTV and iSUV were independent prognostic factors for survival in patients with SCLC of not only intrathoracic malignant hypermetabolic lesions but also of extrathoracic malignant hypermetabolic lesions, while Oh et al. [26] demonstrated that MTV is an independent prognostic factor for survival in patients with SCLC. They used an optimal cutoff of 127 cm^3^ or 64.6 cm^3^ for whole-body tumor MTV, respectively, instead of our 100 cm^3^ for intrathoracic tumor MTV. When those cutoffs of 127 cm^3^ and 64.6 cm^3^ were applied to our study, MTV was also a significant prognostic factor for overall survival in spite of different targets (*P* < 0.001 in both cutoffs).

These studies demonstrated the importance of volumetric PET parameters, such as MTV and TLG, as prognostic factors, which is consistent with our study. However, an important difference is that we investigated only the volumetric PET parameters of intrathoracic malignant hypermetabolic lesions. Autopsy and clinical studies have shown that SCLC can involve multiple sites, including intra-abdominal lesions: liver, adrenal glands, and retroperitoneal lymph nodes, and less frequently, the pancreas, spleen, and kidneys (incidence at presentation = 35%); bone (incidence at presentation = 27–41%); bone marrow (incidence at presentation = 15–30%); brain (incidence at presentation = 10–14%); and subcutaneous soft tissues [28,30], which usually show variable physiological FDG uptake [31]. Therefore, it may be challenging to accurately measure volumetric PET parameters of extrathoracic malignant hypermetabolic lesions using a threshold-based cutoff SUV for delineating the boundaries of the lesions. On the other hand, it is relatively easy to accurately measure volume-based PET parameters of intrathoracic lesions using a cutoff SUV of liver activity. In addition, the measurement of PET parameters including all malignant hypermetabolic lesions may be time-consuming and not available in routine clinical practice. Therefore, it would be advantageous if the PET parameters from intrathoracic lesions only could provide useful prognostic information. The results of our study support this hypothesis. Our results suggest that the volumetric PET parameters of FDG-PET/CT have an important role in predicting prognosis and making risk-adapted therapeutic decisions in patients with SCLC. For example, in a higher MTV group expecting worse prognosis, closer clinical follow-up for surveillance and participation in clinical trials may be considered.

A standardized method for determining SUV thresholds to estimate tumor boundaries has not yet been established. We used a relative SUV threshold for determining the tumor boundary, SUV_avg_ of the liver plus the 2 SDs of each patient, which is different from recent reports [19,26]. One study used a single, fixed SUV threshold of 2.5, while another applied a single, fixed SUV threshold of 3.0. SUV can be affected by many factors, including tumor characteristics, VOI definition, partial volume effect, image resolution, reconstruction methods, noise, time between tracer injection and imaging, attenuation correction, normalization factor, and plasma glucose level [29,32]. Therefore, we presumed that using the same fixed threshold SUV for different patients might have limitations. Comparing tumor activity to background activity is an attractive way to minimize variability and to potentially ensure the quality of scans from test to retest. A variety of backgrounds have been used, including the thighs, back muscles, liver, and mediastinum. Paquet et al. [33] showed that liver SUV is stable over time, when measured centrally as a mean on a single slice in the right lobe of the liver. Thus, we used a relative threshold of SUV for each patient according to the SUV of the liver, which is similar to a previous study [25]. In addition, in the PET Response Criteria in Solid Tumors framework, the SUV of the liver is used as the reference organ [28].

The present study has several limitations. First, because it was a retrospective study, some medical records, such as serum albumin and LDH, were missing in some patients, and it was also difficult to evaluate accurate performance status at the initial stage. Second, not all concerned intrathoracic hypermetabolic lesions were histopathologically confirmed, but the findings of follow-up studies were used for confirming malignancy. Third, a possible limitation was the use of two different kinds of scanners and acquisition protocols, and the accompanying uncertainty of whether or not the volumetric PET parameters were consistent between the two scanners. However, there were no significant differences in demographics, clinical indicators, and disease severity of patients according to the scanner type. For the survival analysis, when applying the same cutoffs of metabolic parameters for primary tumors in subgroups according to scanner type, both sum of MTV and sum of TLG were significant prognostic factors in all subgroups like all patients group. Therefore, we suggest that this issue may not significantly affect our results. Finally, the present study lacks a 2-cohort cross-validation for the cutoff values of quantitative PET prognostic biomarkers to avoid bias, due to the relatively small number of subjects. Therefore, this is a kind of exploratory study.

## Conclusions

In conclusion, the sums of MTV and TLG of the intrathoracic malignant hypermetabolic lesions, volumetric metabolic parameters of FDG-PET/CT, are important independent prognostic factors for survival in addition to age and clinical stage. Volumetric metabolic parameters may be more useful as prognostic factors in patients with SCLC of LD rather than of ED. However, a further, well-designed, confirmatory validation study with a standardized protocol is necessary.

## Competing interests

This study was supported by a grant from the National R&D Program for Cancer Control, Ministry of Health and Welfare, Republic of Korea (#1120150).

## Authors’ contributions

All authors carried out the study concepts and design, literature research, data analysis, study analysis, manuscript preparation and editing. All authors read and approved the final manuscript.
